# Food flora in 17th century northeast region of Brazil in *Historia Naturalis Brasiliae*

**DOI:** 10.1186/1746-4269-10-50

**Published:** 2014-06-25

**Authors:** Maria Franco Trindade Medeiros, Ulysses Paulino Albuquerque

**Affiliations:** 1 Universidade Federal de Campina Grande, Campus de Cuité, Centro de Educação e Saúde, Unidade Acadêmica de Educação, Departamento de Ciências Biológicas, Olho D´Água da Bica s/n, Cuité, Paraíba CEP: 58175-000, Brazil; 2 Laboratory of Applied and Theoretical Ethnobiology (LEA), Department of Biology, Area of Botany, Federal Rural University of Pernambuco, Av. Dom Manoel de Medeiros s/n, Dois Irmãos, Recife, Pernambuco CEP: 52171-900, Brazil

**Keywords:** Food species, Marcgrave, 17th century, Brazil, Historical ethnobotany, Ethnobiology

## Abstract

**Background:**

This article reports historical ethnobotany research conducted from a study of the work *Historia Naturalis Brasiliae* (Natural History of Brazil), authored by Piso and Marcgrave and published in 1648, with main focus on Caatinga of northeast region of Brazil.

**Methods:**

Focusing the content analysis on the section dedicated to plant species with multiple uses, Marcgrave's contribution to the aforementioned work, this research had the following objectives: the retrieval of 17th century knowledge about the food uses of the flora in the northeast region of Brazil, including the taxonomic classifications; the identification of plant parts, their modes of consumption and the ethnic group of consumers; and the verification of the use of these species over time.

**Results:**

The use of 80 food species at the time of the publication of the work is indicated, some of which are endemic to the Caatinga, such as “umbu” (*Spondias tuberosa* Arruda), “mandacaru” (*Cereus jamacaru* DC.) and “carnauba” (*Copernicia cerifera* Mart.). It is noticeable that among the species listed by Marcgrave, some species still lack current studies indicating their real nutritional value. The present study is an unprecedented work because it introduces, in a systematic way, the food plants described in a study of 17th century Brazil.

**Conclusions:**

Finally, this study makes information about plants consumed in the past accessible, aiming to provide material for studies that could develop new food products today.

## Background

Researches based on historical sources of the food plants used on the past by the humanity is a challenge, especially when we are talking about the American’s History, because the sources from this continent are scattered and scarce. Therefore, the ethnobotanical research with its historical point of view starts from the past sources in order to have an actual interpretation of the historical documents.

In recent years, food plants have been addressed in ethnobotanical research, focusing on the factors that influence the knowledge of human populations regarding these resources, but not properly utilizing these historical sources. In semiarid region of South America, aspects such as modes of cultural transmission of knowledge and the influence of ecological variables on the use of plants, including the time spent in the gathering process, soil deterioration due to grazing and nutritional constituents, have been identified and investigated as important factors in this dynamic of knowledge associated to food plants, especially on the semiarid region of Argentina see for example [1-8].

Particularly regarding Brazil, among the small number of studies devoted to the record of the knowledge and use of food plants today, those developed by [9-16] can be mentioned. There are historical studies of plants used for food, such as those conducted by [17-19], addressing the issue of emergency food to combat hunger in the Caatinga region (Northeast Brazil). The lack of information about food plants in the past is attributed to the methodological difficulty in accessing historical documents. Nowadays these methodological issues comes from the difficulty in accessing these specific information of the local population [20] because these persons underutilize the food plants of certain environments, considering the plants edible only in subsistence situations [21]. In addition, plants used on an emergency basis are characterised as being of low nutritional quality and having an unpleasant taste [22], which contributes to maintaining a standard of local consumption that is restricted to species from environments that present hostile natural conditions.

Currently, there are few published Brazilian ethnobotanical works and particularly few that take historical documents as records of the knowledge of food flora in past centuries. Given this context, it becomes obvious that there is a lack of knowledge on the food potential of Brazilian flora species, not only in the present moment but also in the past, when it is considered all the richness and potential of the Brazilian flora to be researched and valued. In this field the historical analysis still needs a more strong support to develop researches. The present research has been developed to fill this absence of works on this theme and to identify a great source of documents for accessing information on the knowledge of the food flora of Brazil.

As a source of historical research on the flora of the Brazilian Northeastern Region, the extensive contribution by Guilherme Piso and Jorge Marcgrave, titled *Historia Naturalis Brasiliae* (Natural History of Brazil), published for the first time in 1648, is mentioned [23]. This work is a milestone, and it represents a truly scientific effort to make the flora and fauna of Brazil known [24]. Piso's research concentrated on the medicinal applications of plant species. Marcgrave took as his main work a more comprehensive inquiry into botanical and zoological studies. In his writings, Marcgrave discusses in detail the useful plants of the northeast region of Brazil.

Taking this 17th century written source as a reference, the present study had the following objectives: 1. to retrieve the knowledge of food plants recorded by Marcgrave in the 17th century, updating the taxonomic identification of the reported species and verifying their presence in contemporary food practice; 2. to identify the parts of the plant used as a food resource as well as the mode of consumption of these species and determine whether there was any relation between these aspects with respect to mitigating and/or removing any undesirable effects of the vegetative organ consumed; and 3. to identify the ethnic groups mentioned by Marcgrave as consumers of these plants. The present study is an unprecedented work because it introduces, in a systematic way, the food plants described in 17th century Brazil. Finally, this study increases the accessibility of information about plants consumed in the past, aiming to provide context for studies of potential new food products today.

### Brief biographical review of the author - Jorge Marcgrave

Through the writings of Moreira and Urban [25,26], it is possible to illuminate the life of Marcgrave. Of German origin, he was born in Liebstadt, in Saxony, on September 20, 1610. At 17 years of age, he joined the University of Lipsi and several others, where for six years he devoted himself to the study of mathematics, medicine and natural sciences. He became an astronomer, chemist, physician, naturalist, geographer and architect.

The arrival of Marcgrave in Brazil was associated with the activities conducted by the West India Company, which operated through a monopoly granted by the Dutch government as an overseas offensive, aiming to destroy the colonial bases of Iberian wealth and power [27]. Founded in 1621, the Company followed the institutional model for the Americas and the western coast of Africa that was adopted by the East India Company in Asia from the early years of the 17th century. Through this model, the Company promoted trade and colonisation in the mentioned regions, at the expense of the Spanish and Portuguese presence [27,28].

Brazil also became a Dutch target due to a series of contingencies, including profits from sugar and Brazilwood, as well as its geographically strategic position, particularly the northeast, as an operations base against the Spanish and Portuguese fleet [28,29]. Portuguese groups that were established in Amsterdam to escape the persecution of the Inquisition in the Iberian Peninsula controlled the northeast sugar trade [28]. These Portuguese living in Amsterdam had been involved in the financing of the sugar agroindustry and the commercialisation of the product in European markets since the beginning of Northeast Brazil's colonisation [30]. These contacts with the Portuguese allowed the Dutch to know the economic and social conditions of the northeast coast, its ports and even the urban environment of the city of Olinda (in Pernambuco, NE Brazil). This connection was vital for executing attacks in Bahia, followed by those against Pernambuco.

In 1630, the army of the West India Company had already conquered Olinda and Recife (in Pernambuco). Seven years later, in 1637, Count João Maurício de Nassau assumed the government of Dutch Brazil, staying in Brazilian lands until 1644. Transferring the political centre from Olinda to Recife, Nassau assumed control over the region as governor-general [31]. This Dutch occupation of Northeast Brazil extended until the year 1654, when the Dutch were expelled by insurrection movements in Pernambuco [28].

Before leaving for Brazil, Nassau expressed to João de Laet, one of the best chroniclers of the West India Company, the desire for a scientific expedition to be organised to the domains that the Netherlands intended to settle in Brazil. For this reason, the Dutch physician and naturalist Guilherme Piso was appointed chief of the first strictly scientific mission issued by an European country in the lands of the “new world” [32]. In response to the order for a new doctor, which came from the administrative council in Pernambuco, Piso had been appointed by the Company as a doctor to come to Brazil to replace Dr. Guilherme van Millaene, Nassau's physician, who had died in 1637 [25]. Therefore, Piso would continue to implement a scientific mission in Brazil, with the aid of two trainees of medicine and mathematics, Cralitz and Marcgrave, in addition to a German painter who would illustrate their work, who remains anonymous today [32-34].

Marcgrave was well recommended by Laet, to whom he was close in the university circle, when Guilherme Piso invited him to join the expedition to Brazil. Together with Piso, Marcgrave was hired as an astronomer for this expedition [25,35].

At 28 years of age, Marcgrave left the Netherlands in January 1st, 1638, destined for the city of São Salvador, in Bahia. The scientific expedition embarked on the fleet established to reinforce the squad of Mauricio de Nassau to attack Bahia [25,32].

**Figure 1:**
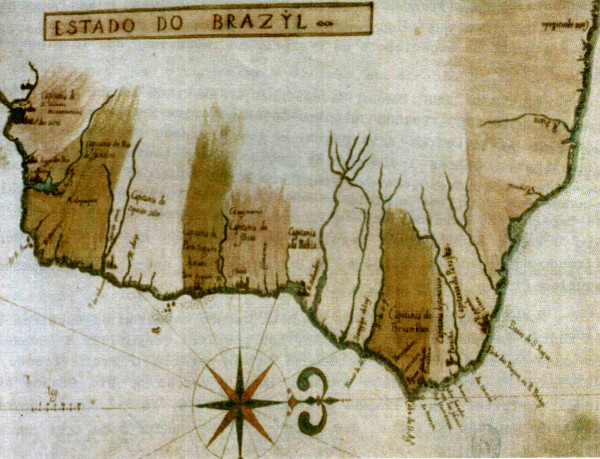
**Map from the coast of Brazil, showing the Northeast coast at the left, where Margrave undertook his scientific action **[38]**.**

In Bahia, Marcgrave made topographical maps and toured the coast collecting vegetable and animal specimens. Following the team to Pernambuco, Marcgrave undertook much of his scientific action in this region. Marcgrave was in Brazil for six years, where he travelled from the São Francisco River to the State of Ceará, also staying in the states of Maranhão, Sergipe and Alagoas [36,37] (see Figure 1).

Although Marcgrave had planned his return to Europe for the year 1644, at this same time, he received orders from Mauricio de Nassau to proceed to Africa and undertake a scientific expedition similar to that conducted in Brazil [25,32]. In the preface of the first edition of *Historia Naturalis Brasiliae*, Laet reports that Marcgrave's trip to Brazil was marked by the tireless investigation of “new things”. Marcgrave gave his herbarium to the Count of Nassau and went to Africa, dying shortly after arriving there, in July or August 1644 at 34 years of age.

### *The historical source:* Historia Naturalis Brasiliae

Originally published in 1648, written by Guilherme Piso and co-authored by Jorge Marcgrave, *Historia Naturalis Brasiliae* is considered the Americas' first scientific treatise and, with regard to Brazil, the first report of the flora and the first medical botany of Brazil [35]. Piso and Marcgrave are thus considered the founders of the studies of nosology and natural history in Brazil [25]. *Historia Naturalis Brasiliae* described plant species that were only studied again 200 years after this scientific effort. Several authors have worked on the identification of these plants and/or cited information presented by Piso and Marcgrave, e.g., [39-42].

The original version is in the form of an *in folio* volume with discontinuous paging, written in Latin and published by Oficina Elsevier, and presents an inscription by Piso and Marcgrave to Count John Maurice of Nassau, governor of Dutch Brazil. The local flora, especially in the State of Pernambuco, constitute the majority of the presented information. This area was the region where Marcgrave and Piso worked during most of their stay in Brazil. Urban [26] states that the authors worked in Pernambuco between the years 1640 and 1644, with few journeys to other states.

The first treatise of the work is authored by Piso and is titled *De Medicina Brasiliensis*, comprising four books dedicated to Brazilian tropical medicine, with the citation of 89 plants. After this treatise, Marcgrave's *Historiae rerum Naturalium Brasiliae* is presented*,* which was written by Laet, who also added additional information. Laet took charge of publishing this work and commented on Marcgrave's writings, stating that the original had been written in encrypted letters (cipher alphabet, Marcgrave's own invention) to protect the manuscripts and that the original "should be previously deciphered and transcribed with greater labour than someone would have the courage to take upon himself” [23]. Marcgrave's entire treatise includes eight books. The first three books contain the *herbs*, *bushes* and *trees*, making references to 279 plants. The other books contain zoology and provide astronomical, ethnographic and geographical comments.

The second edition, published 10 years after *Historia Naturalis Brasiliae*[23], is titled *De Indiae utriusque re Naturali et Medica* (1658) [43]. In this edition, only the plants presented in Piso's treatise are included, along with Marcgrave's writings on the geography, meteorology and ethnography of the region. Piso mentions 26 plants in addition to those previously considered.

For the present research on food plants, the Portuguese language version of the first edition of *Historia Naturalis Brasiliae* was used. In his treatise, Marcgrave not only considers medicinal plants, as Piso does, but also records industrial and home uses and curious properties of the studied organisms. Marcgrave collected specimens, sought to describe them *in loco,* prepared illustrations (drawings and watercolours) and gathered these “curious things” to his cabinet, creating a herbarium and a museum of “curious” objects. The Illustrations of the plants in the first edition are mostly his own, including the book’s ornamentation [23]. Laet, while organising the edition of the work, ordered 14 drawings of Marcgrave's herbarium material and considers not only this set of illustrations but also those made by Marcgrave, distributing these prints throughout the text in a confusing way for the reader.

## Methods

### Collection and analysis of historical ethnobotanical data

The edition of *Historia Naturalis Brasiliae* published by Companhia Editora Nacional (Brazil) consists of two volumes published at different times: the first one, with Marcgrave's writings, published in 1942 and translated by José Procópio de Magalhães [36], and the second one, providing Piso's information, published in 1948 with a translation by Alexandre Correia. The work on this Portuguese version was located and consulted in the Blanche Knopf Central Library of the Joaquim Nabuco Foundation (FUNDAJ/Recife). Because the present study pertained to food plants, the effort focused on the study of Marcgrave's writings; therefore, the first book was used.

The study of Marcgrave's work was performed in two reading stages. First, a pre-reading of the source material was performed to establish an initial familiarity with the work and to designate the items that would compose the data collection chart. Subsequently, an in-depth reading was performed, with the concomitant transcription of information regarding food plants described by Marcgrave, opting to keep the palaeographic form of the text [44].

With the collected data, a database was created containing the popular names, scientific names, indicated food use, consumed part of the plant, mode of consumption, ethnic groups that used the plant as food, plant habit and whether there was any contraindication.

The current identification of the plants is hampered by the impossibility of consulting Marcgrave's and Piso's collections. Marcgrave's and Piso's herbaria were lost [35], and all that remains are some of Marcgrave's illustrations and other prints that Laet, organiser of the first edition, ordered to be made directly from the collected specimens. Because direct observation of the botanical material collected by Marcgrave was not possible, the scientific names reported in the work were updated and considered taxonomic tracks. The revision work conducted by Pickel and organised by [45] was adopted for an update of the identification of these Marcgravian plants. For each scientific name present in this study by Pickel, a taxonomic update was sought through consultation with the literature, the botany experts and databases of the Missouri Botanical Garden’s VAST (VAScular Tropicos) nomenclatural database W3 Tropicos [46] and The International Plant Names Index (IPNI) [47].

The consultation of the recently mentioned revision by [45] of Piso's and Marcgrave's writings also intended to verify whether Piso mentioned the food use of those plants if the data presented by Marcgrave did not include this information. If there was an indication of food use, this additional information on Marcgravian plants was also incorporated into this work.

For historical contextualisation and relation to the life of Marcgrave and to verify the presence of the food plants mentioned by Marcgrave in contemporary food practice the following material relating to the period of Dutch rule in Brazil and to studies that included the relevant topics was consulted: food plants in a general manner, food plants present in ethnobotanical studies conducted in the Brazilian Northeast and assessments of the nutritional components of the food plant species mentioned in *Historia Naturalis Brasiliae*. The collection accessed is deposited at the National Library Foundation of Rio de Janeiro, the Marine Library São Paulo de Vasconcellos, from the Institute of Philosophy and Social Sciences of the Federal University of Rio de Janeiro (IFCS/UFRJ), and the Library of the National Museum of the Federal University of Rio de Janeiro (MN/UFRJ). Additionally, a search was performed for scientific works in the JSTOR (http://www.jstor.org), Scopus (http://www.scopus.com) and Scirus (http://www.scirus.com) databases in addition to specialised journals, using the keywords “etnobotânica” [ethnobotany] AND “plantas alimentícias” [food plants], as well as “etnobotânic” [ethnobotany] AND “Nordeste do Brasil” [Northeast of Brazil]. For research in ethnobotany, articles published in journals during the period from 2007 to 2013 were analysed. In these same databases, a survey was conducted for each species and popular name in the studied work.

## Results and discussion

### Food plants referenced by Marcgrave

Of the 279 plants mentioned in *Historia Naturalis Brasiliae*, 85 (23%) had an indication of food use reported by Marcgrave [36] (Table 1). Among these food plants, it is possible to identify the taxonomic tracks of 80 species, contained within 74 genera and 44 families. For the term “herb”, it was not possible to identify a taxonomic track because “herb” does not have an identification presented by Marcgrave himself (“*quam Auctor non nominat*”, [23], p. 19) (Table 1). For two of the species displayed in the Table 1, actual botanical information linked to their popular names was kept separated. This form of data organisation has different causes. In the case of one of these two species, although the plants popularly designated by “nhua” and “pitomba” are treated separately by Marcgrave, Pickel [45] states that their descriptions are identical and would refer to the same species, identified as *Talisia esculenta* (A. St.- Hil.) Radlk. (Sapindaceae) (Table 1). The other species case was related to the terms “araticu ponhe” and “araticum apê” corresponded to two different species of Annonaceae, which are currently synonymous with *Annona montana* Macfad (Table 1).

**Table 1:** **Food species mentioned by Marcgrave in the work *Historia Naturalis Brasiliae*** [36]

**Taxonomic track**	**Popular name**	**H**	**PU**	**MC**	**WU**	**CI**	**Report of the naturalist**
**Amaranthaceae**							
*Amaranthus viridis* L.	Cararu; Bredos	H	x	Cooked	x	x	This herb is cooked as a vegetable in the same manner as chard, has a good taste and easily softens when cooked (HNB, p. 13)
*Iresine vermicularis* (L.) Moq.	Perexil	H	Le; Bar	Cooked and seasoned, served with beef and fish	Portuguese	x	The leaves and branches, cut short and cooked with a little vinegar, can be seasoned and preserved as a pickle to be eaten with beef and fish. These parts have great flavour and are highly valued by the Portuguese; they increase appetite, develop urine and open the oppilation of the viscera (HNB, p 14.)
**Anacardiaceae**							
*Anacardium occidentale* L.	Acaiaiba; Acaiuiba	Tr	Nu; Fr	As wine and fresh	Indians	x	The Indians appreciate more the nut for food than this fruit, from which they extract a wine (HNB, p. 94–95)
*Schinus terebinthifolius* Raddi	Aroeira	Tr	Fr	As wine, vinegar and honey	Americans	Dries the intestine	From this fruit cooked in water, according to the decoction mode, a very good wine or potion, vinegar or honey are made (HNB, p. 90–91)
*Spondias purpurea* L.	Acaia; Ibametara	Tr	Le	As spice	x	x	From the crushed new leaves, a seasoning of very pleasant flavour to roasted meats is made (HNB, p. 129)
*Spondias tuberosa* Arruda*	Umbú	Tr	Fr; Le; Br	As beverage or fresh	x	x	The ripe fruit has a nice, bittersweet flavour, used like the leaves, i.e., as a beverage. When chewed, the root crumbles into a watery, fresh and palatable juice, being used by weary travellers as an admirable refreshment, resembling the "watermelon" regarding the sweetness and wholesomeness of water (HNB-M., p. 108; HNB-P., p. 77)
*Tapirira guianensis* Aubl.	Copiiba	Tr	Fr	Fresh	Indians	x	The fruit is eaten by sucking the juice and discarding the skin (HNB, p. 121)
**Annonaceae**							
*Annona montana* Macfad.	Araticu ponhe	Tr	Fr	x	x	x	The fruit is not edible unless it has fallen spontaneously because it is then soft as porridge; the pulp resembles a mass of leavened bread, to which a little honey has been mixed, and tastes sweet and tangy spicy (HNB, p. 93)
	Araticum apê*	Tr	Fr	X	x	x	Acid-sweet, edible fruit, but wild and cold and therefore not craved by all people (HNB-M, p. 94; HNB-P, p. 70; 142)
*Xylopia frutescens* Aubl.	Ibira	Tr	Fr	Dried, as pepper	x	x	Its fruit is oval with the size of a hazelnut, with an aromatic and spicy taste; used dried and reduced, it can substitute for pepper (HNB, p. 99–100)
**Apocynaceae**							
*Hancornia speciosa* Gomes	Mangabiba; Mangaiba	Tr	Fr; S	Fruit and seeds are eaten together	x	Fruits on the plant are impregnated with an acrid and bitter latex	The fruits are not edible unless they fall from the tree spontaneously; its pulp is soft as butter and has a very nice and acidic taste, with seeds of albumen sweet flavour (HNB, p. 121–123)
**Araceae**							
*Colocasia esculenta* (L.) Schott	Taiaoba	H	R	Cooked	x	X	Its root is eaten cooked like the potato; it is sweet, with a remarkable flavour, similar to musk or violet (HNB, p. 36)
*Montrichardia linifera* (Arruda) Schott	Aniga Iba	Tr	Fr	X	Indians	X	This fruit is eaten in case of need; it is eaten in times of hunger (HNB, p. 106)
**Arecaceae**							
*Attalea oleifera* Barb. Rodr.	Pindoba	Tr	Fr	The pulp is eaten with flour	Blacks	X	It is eaten with flour by the blacks (HNB, p. 133–134)
*Cocos nucifera* L.	Inaia guacuiba; Coqueiro	Tr	Fr; B	The core of the ripe fruit is eaten and the water is drunk; the milk extracted from the core of the fruit is cooked with rice for dessert; the bulb is eaten. Honey, sugar, vinegar and wine are also made	x	The wine is harmful for the hydropics and those who have obstructed spleen	The cavity is filled with a very pleasant water to drink; it is sweet, cold and clear (HNB, p. 138–141)
*Copernicia cerifera* Mart.*	Carana iba; Anana chi carirí	Tr	Fr	Fresh	x		Sweet after ripe (HNB-M, p. 62; HNB-P, p. 62)
*Syagrus coronata* (Mart.) Becc.	Urucuri iba	Tr	S	X	x	X	Inside the fruit, there is a hard seed; an edible white nut is found (HNB, p. 104)
**Bignoniaceae**							
*Crescentia cujete* L.	Cuiete; Cochine	Tr	Fr	The pulp is edible	x	X	The unripe fruit encloses a white juicy pulp, with a smell close to that of watercress, slightly sweet. The barbarians eat this fruit in case of necessity (HNB, p. 123)
**Bixaceae**							
*Bixa orellana* L.	Urucu	Tr	S	The seeds are processed into a paste, which is mixed with manioc pap	Indians	x	The paste of urucu has a good taste and is aromatic but tastes a little bitter, being eaten with a porridge of manioc called carimã (HNB, p. 61)
**Bromeliaceae**							
*Ananas sativus* Schult. & Schult. f.	Nana; Ananas	H	Fr	Fresh and in conserved with sugar	Indians	X	The fruit has the sweetest smell and very pleasant flavour, like strawberries, extremely juicy (HNB, p. 33)
*Bromelia karatas* L.	Nana brava; Caraguata-acanga	Bu	Fr	X	x	X	Produces an edible fruit, with a length equivalent to five fingers (HNB, p. 88)
**Cactaceae**							
*Cereus jamacaru* D.C.*	Iamacarú; Cardon; Caxambú	Tr	Fr	Fresh	x	X	Edible fruit (HNB-M, p. 126; HNB-P, p. 99)
*Hylocereus undatus* (Haw.) Britton & Rose	Iamacarú	H	Fr	Fresh	x	x	Fruit with succulent, tasty flesh, filled with black seeds. The whole internal part is eaten (HNB, p. 23–24)
*Opuntia brasiliensis* (Willd.) Haw.	Iamacarú	Tr	Fr	The fruit and the grains are edible	x	Dries the stomach and provokes flatulence	It is said that the fruit, when eaten with the grain, dries the stomach; it provides good and pleasant nourishment (HNB, p. 126–127)
**Cannaceae**							
*Canna indica* L.	Meeru	H	R	X	Blacks	X	The blacks eat the root (HNB, p. 4)
**Capparaceae**							
*Crataeva tapia* L.	Tapiá	Tr	Fr	X	x	X	The fruit is edible (HNB, p. 98)
**Caricaceae**							
*Carica papaya* L.	Mamoeira; Papay; Mamão	Tr	Fr	Raw or cooked	x	x	The fruit can be eaten raw but is usually eaten cooked alone or mixed with meat (HNB, p. 102–104)
*Jaracatia* sp.	Iaracatiá	Tr	Fr	Raw or cooked	x	X	When ripe, falls spontaneously and is eaten raw or cooked (HNB, p. 128–129)
**Chrysobalanaceae**							
*Chrysobalanus icaco* L.	Guaieru; Guajeru	Bu	Fr	X	x	X	This fruit has sweet white flesh; it is edible (HNB, p. 77)
*Couepia rufa* Ducke	Guitiiba	Tr	Fr	X	Indians	x	The pulp is eaten, but not the seed; the pulp is negligible and gives the impression of having sand between the teeth when chewed but has a sweet taste and good smell, reminiscent of bread that has been recently made (HNB, p. 114)
**Cleomaceae**							
*Cleome rosea* Valh ex DC.	Micambe de Angola	H	S	X	Blacks	X	Used by blacks as food (HNB, p. 10)
**Clusiaceae**							
*Clusia nemorosa* G. Mey.	Coapoiba; Pao gamelo	Tr	Fr	x	x	X	They are eaten by some but are not much appreciated (HNB, p. 131–132)
*Platonia insignis* Mart.	Ibacuri-pari	Tr	Fr	The pulp is edible	x	X	The pulp of the fruit has an acrid and a slightly bitter taste but is edible (HNB, p. 119)
*Rheedia macrophylla* (Mart.) Planch. & Triana	Ibacuru-pari	Tr	Nu	X	Indians	X	The albumen, which is very white, is edible (HNB, p. 119–120)
**Convolvulaceae**							
*Ipomoea batatas* (L.) Lam.	Ietica; Quiquoaquianputu; Batata	H	Po	Cooked, roasted, as fermented drink	Indians	x	They are steamed or roasted in ashes and have a great flavour, more preferable than the radish. The fresh potato, when crushed and macerated in a little water, provides a drink (HNB, p. 16–17)
**Cucurbitaceae**							
*Citrullus lanatus* (Thunb.) Matsum. & Nakai	Jaee; Balancia	H	Fr	The fresh pulp is eaten, and the water is drunk	x	X	It has a juiciest pulp of good flavour; it has such a large amount of sweet and cold water that, during the meal, it may be taken as if it was in a glass (HNB, p. 22)
*Cucumis* sp.	Pepino Silvestre do Brasil	H	Fr	X	x	X	It is edible (HNB, p. 44)
*Cucurbita pepo* L.	Iurum; Bóbora; Pompoen	H	Fr	Roasted or cooked	x	X	The boiled or baked fruit in the ashes has a good taste (HNB, p. 44)
**Dioscoreaceae**							
*Polynome alata* (L.) Salisb.	Cará; Inhame de São Thomé; Quiquoaquicongo	H	R	Cooked or dried	Inhabitants from Guinea	X	The root, when cooked with butter or olive oil and pepper, has a great flavour; it is dry and floury, and thus, the people from Guinea eat it to replace bread (HNB, p. 29)
**Euphorbiaceae**							
*Manihot esculenta* Crantz	Maniiba; Mandijba; Mandioca	Bu	R; Le	For preparing flour, pap, bread, cakes	Indians	The milky and glutinous juice of the root kills all living beings	The leaves, when pounded and cooked with oil or butter, are edible (HNB, p. 65–67)
*Manihot glaziovii* Müll. Arg.	Maniçoba; Mandijba	Tr	Le	Cooked	x	X	The leaves, well crushed with a pestle in a wood mortar and then cooked with olive oil and butter, are eaten like cooked spinach (HNB, p. 68)
**Fabaceae**							
*Arachis hypogaea* L.	Mundubi	H	R	Cooked	x	Eaten in a large amount cause headaches	Are served to eat cooked and presented as dessert (HNB, p. 37)
*Cajanus cajan* (L.) Huth	Comanda guira	Bu	S	Cooked	Indians	Laxative	Has a good taste when cooked (HNB, p. 62)
*Geoffraea* sp.	Umari	Tr	Fr	Cooked	x	The unripe fruit is harmful for the stomach	Eaten unripe is harmful to the stomach and causes vomiting, so it is usually cooked and mashed with the seeds in the mortar, and the paste is eaten replacing bread or flour in dishes of beef and fish (HNB, p. 121)
*Hymenaea martiana* Hayne	Jetaiba	Tr	Fr	X	Indians	X	The flesh, whose taste is not disregarded, is eaten (HNB, p. 101)
*Inga vera* Willd.	Inga	Tr	Fr	Fresh	Indians	X	This edible fruit is palatable (HNB, p. 111)
*Lablab purpureus* (L.) Sweet	Mandatia	H	S	Cooked	x	X	The seeds are edible, with an excellent taste if mixed with spices and cooked (HNB, p. 52)
*Phyllocalyx edulis* O. Berg.	Ibiruba	Tr	Fr	x	x	x	The fruit has a juicy pulp, with an acidic taste and slowly embittering, not unpleasant, and has a sweet and grapey smell; the seed is discarded, the rest is eaten; it is an excellent fruit and can be eaten in a large amount without inconvenience (HNB, p. 132)
*Voandzeia subterranea* (L.) DC.	Mandubi d'Angola	H	R	Roasted	x	X	Edible roots (HNB, p. 43–44)
**Heliconiaceae**							
*Heliconia* vaginalis Benth.	Aglutiguepo-obi; Acutitiguepo; Cotitepooba	H	R	Roasted and cooked	x	X	The root is roasted or boiled for food in times of hunger (HNB, p. 53)
**Lamiaceae**							
*Vitex rufescens* A. Juss.	Ibapurunga	Tr	Fr	Fresh	Indians	X	These fruits are eaten without the bark; they are sweet but not too manifest (HNB, p. 116)
**Lecythidaceae**							
*Lecythis pisonis* Cambess.	Iaçapucaya	Tr	Nu	Raw and roasted	x	X	The nuts have an albumen with great flavour, which is eaten raw or roasted (HNB, p. 128)
**Malpighiaceae**							
*Byrsonima* sp.	Mureci	Tr	Fr	X	Indians	X	The fruit of this tree consists of berries with the figure and size of briar fruits and are edible (HNB, p. 118)
**Malvaceae**							
*Hibiscus esculentus* L.	Quingombo; Quillombo	H	Fr	Cooked	x	x	This pericarp smells like pods when green and have a sweetish taste; it is entirely cooked in water and is eaten cooked with olive oil, vinegar and pepper, the more ripe, the better to cook (HNB, p. 31)
**Marantaceae**							
*Saranthe marcgravii* Pickel	Tamoatarana	H	B	Cooked	x	X	It is cooked and eaten like (sweet) potatoes; it has a good flavour (HNB, p. 53–54)
**Melastomataceae**							
*Clidemia hirta* (L.) D. Don	Caaghiyuyo	Bu	Fr	Fresh or as juice	Ethiopians	X	Fruits, with a sweet taste, are eaten by the Ethiopians and provide a juice more or less like the blueberry (HNB, p. 59)
*Mouriri pusa* Gardner ex Gardner	Curuiri	Tr	S	X	Indians	X	It is edible and often enjoyable (HNB, p. 109–110)
**Moraceae**							
*Maclura tinctoria* (L.) D. Don ex Steud.	Tataiiba	Tr	Fr	Fresh or with sugar or wine	x	x	The fruits are juicy and sweet and are eaten as blackberries, pure or with sugar and wine (HNB, p. 119)
**Musaceae**							
*Musa paradisiaca* L.	Pacoeira; Quibuaaquitiba	Tr	Fr	Fresh, cooked or fried	x	X	It has a good flavour and is eaten pure, with manioc flour, baked or fried in olive oil or butter (HNB, p. 137–138)
**Myrtaceae**							
*Campomanesia dichotoma* (O. Berg) Mattos	Ibabiraba	Tr	Fr; S	X	Indians	X	Its pulp and seeds are eaten together; the taste is sweet, somewhat mixed with resin (HNB, p. 117)
*Eugenia uniflora* L.*	Ibipitanga; Ibipitinga; Ubapitanga	Tr	Fr	x	x	x	Very juicy fruit with red pulp and a hot taste, with a bit of pepper; it is an attractive dessert (HNB-M, p. 116; HNB-P, p. 121)
*Psidium guineense* Sw.	Araça-iba	Bu	Fr	In conserve with sugar (marmalade)	x	X	It tastes good, sweet and astringent (HNB, p. 62)
*Psidium guajava* L.	Guayaba; Granaet-peeren	Tr	Fr; S	Cooked and raw	x	It is laxative when ingested, being thus unhealthy if eaten excessively	The pulp contains small seeds, which are eaten together; the fruits are small and with a pleasant flavour; it is great both raw and cooked (HNB, p. 104–105)
**Passifloraceae**							
*Passiflora cincinnata* Mast.	Murucujá	Bu	Fr	x	x	X	The fruit is cut transversely when one wants to eat it, being recommended both for its scent and for its taste (HNB, p. 71)
*Passiflora quadrangularis* L.	Murucuia-guaçú; Gauinumbi acaiuba	Bu	Fr; S	The pulp is sucked with the seeds	x	X	The smell and flavour of the fruit are sweet and mild; to eat it, it is cut crosswise, and the pulp is slightly separated from the pericarp (HNB, p. 70)
**Pedaliaceae**							
*Sesamum orientale* L.	Sésamo; Gangila; Girgilim	H	S	Oil extracted from the seed, and residuals eaten with corn	Blacks	X	An oil is produced, which is commonly eaten and used (HNB, p. 21)
**Piperaceae**							
*Piper marginatum* Jacq.	Nhamdu; Betre	Bu	Fr	Dried	x	x	Sun-dried fruits are sour as the best black pepper; it is not a bad food and gives a good flavour (HNB, p. 75)
**Poaceae**							
*Arundo saccharifera* Garsault	Vubae; Tacomaree	Bu	Cu	To sweeten the food (produce sugar)	x	X	The pith of the cane is solid, juicy, sweet and white (HNB, p. 82)
**Portulacaceae**							
*Portulaca oleracea* L.	Caaponga	H	x	Cooked	x	X	This herb is eaten cooked (HNB, p. 49)
**Rubiaceae**							
*Genipa americana* L.	Ianipaba; Ienipapo	Tr	Fr; S	Fresh or as wine	x	X	From the acidic flavoured pulp, refreshing and with a pleasant smell, a wine is squeezed; its grains or seeds are also eaten with the flesh (HNB, p. 92–93)
**Sapindaceae**							
*Talisia esculenta* (A. St.-Hil.) Radlk.	Nhua	Tr	Fr	X	x	x	Fruit has a somewhat bitter taste; when ripe, it falls, being picked up and eaten (HNB, p. 100)
	Pitoma	Tr	Fr	The pulp is eaten	x	X	The flesh tastes astringently acidic and is separated from the bark, cut and eaten (HNB, p. 125)
**Sapotaceae**							
*Pouteria grandiflora* (A. DC.) Baehni	Guiti-toroba; Steen-appel	Tr	Fr	Ripe	Indians	It is inedible before ripe because it is replete with acrid latex	The fruit, when opened, exudes a strong disgusting smell, like old grease, with a sweet tasting pulp; the fruit is edible (HNB, p. 113–114)
**Solanaceae**							
*Capsicum annuum* L.	Quiya uçu; Pimenta grande; Pimentões	H	Fr	As spice	Indians	x	The Indians smash this pepper with salt and call this mixture Iuquitayae, with which they season the food at the time of the meal in the same way that we use salt (HNB, p. 39)
*Capsicum annuum* var. *frutescens* (L.) Kuntze	Quiya cumari; Quiyaqui; Pimenta malagueta	H	Fr	X	Indians	x	This fruit tastes very bitter, much spicier than the other species (HNB, p. 39)
*Physalis peruviana* L.	Camarú	H	Fr	x	x	x	The fruit is edible and has a flavour similar to our bladder cherry (HNB, p. 12)
*Solanum agrarium* Sendtn.	Iuati	Bu	Fr	X	x	x	Edible fruit like gooseberry; presents a pleasant acidic taste (HNB, p. 80)
*Solanum melongena* L.	Belingela; Macumba; Tongu	H	Fr	Cooked	x	x	This fruit is baked seasoned with olive oil and pepper and has the flavour of lemon (HNB, p. 24)
**Talinaceae**							
*Talinum paniculatum* (Jacq.) Gaertn.	Acetosa	H	x	Used in salads	x	x	It has a nice acidity; it is used for salads (HNB, p. 23)
**Urticaceae**							
*Cecropia concolor* Willd.	Ambaiba	Tr	Fr	X	Indians	x	Are taken as teeth and eaten (HNB, p. 91–92)
**Xanthorrhoeaceae**							
*Aloe vera* (L.) Burm. f.	Caraguata	H	Le	Cooked	x	x	The leaf and the caudex, cooked in an underground oven, are edible, tasting like diacitrum (HNB, p. 38)
**Ximeniaceae**							
*Ximenia americana* L.	Jua umbu	Tr	Fr	X	Indians	x	This fruit is edible (HNB, p. 108)
**Indeterminada**							
	Erva (o autor não menciona o nome)	H	Le; Fl	X	Blacks from Angola	x	The blacks from Angola eat the leaves and flowers (HNB, p. 19)

Legend: H = habit (H = herb; Bu = bush; Tr = tree); PU = part used (R = root; S = seeds; Fr = fruit; B = bulb; Le = Leave; Cu = culm; Nu = nut; Br = branches; Po = potato; Fl = flower); MC = mode of consumption; WU = who utilises; CI = contraindication; HNB = *Historia Naturalis Brasiliae*; HNB-M = *Historia Naturalis Brasiliae*, Marcgrave´s book; HNB-P = *Historia Naturalis Brasiliae*, Piso´s book; * = species mentioned by Marcgrave but whose food use was discriminated by Piso; x = without information.

The families with the largest numbers of species mentioned were Fabaceae (eight species); Anacardiaceae and Solanaceae (five species each), Arecaceae and Myrtaceae (four species each); and Cactaceae, Clusiaceae and Passifloraceae (three species each). Nevertheless, the above families had not corresponded in their entirely to the genera that include the largest number of species. The most varied genera in terms of species were *Capsicum, Manihot, Passiflora, Psidium, Solanum* and *Spondias*, with two species each. These genera include plants especially recognised by their fruits and appreciated for their taste, with the exception of *Manihot* and/or species used as spices in cooking.

The descriptions of the plants by Marcgrave include a comparison with European plants and aspects related to the habit, the shape and contours of the leaf and the shape of the flowers, fruits and seeds. Often, the entries include quotations from ancient and contemporary authors on botany, including Pliny [23-79], Dioscorides [33,34,36,37,44-90], Avicenna (980-1037), Garcia de la Huerta (1500-1568) and others. This inclusion of information might evidence the author's familiarity with the European flora and literature, as Pickel states [35], but it must also be considered that these inserts could have been authored by Laet while editing and publishing the books in 1648. Laet added comments to Marcgrave's studies, aiming to complement the information presented for each plant. This insertion of information can be proven, for example, in the note presented by Laet for the plant known as “Sesame, Gangila (term from Congo)” (*Sesamum orientale* L., Pedaliaceae), the excerpt for which is transcribed below: “[…] Note. Fray João de Santos in the History of Eastern Ethiopia, b. I chap. 4, states that in all these provinces, sesame is much found, most whitish and excellent, from which an oil is made [… ]” ([36], p. 21) (Table 1). Laet often provides information from other authors in his notes, even indicating the reading of Piso's treatise, present in the work *Historia Naturalis Brasiliae*.

Taking into consideration all the categories of use and respecting the ordering of plants by habit presented in Marcgrave's treatise, the herbs formed the largest group, followed by trees and bushes (with 137, 99 and 43 plants, respectively). If considering anything other than the list of food plants, tree species become the most significant, with 52% (43 plants) of the total, followed by herbs (32%, 27 plants) and bushes (16%, 13 plants). Marcgrave's systematic order of plants follows the division into herbs, bushes and trees. This organisation presented by the author is based on the artificial classification systems. Influenced by the philosophical premises concerning the principle of immutability of species, these classification systems aimed to be an efficient way of identifying samples without needing to show affinity relations between species [48,49]. Marcgrave establishes an ordering of the plants he considered, similar to the classification proposed during the 17th century by Andrea Caesalpino (1519-1603), who established one of the first classifications based on criteria of similarity and defined levels of taxonomic hierarchy.

### Parts of the plant used, mode of preparation and consumption of the food resources and ethnic group of consumers

In addition to the vegetable organs of root, leaf, flower, fruit and seed, Marcgrave also used other terms to describe the parts of the plant used as food (Table 1). There are references to the consumption of the potato, bulb, stem, nut, branches and grains. From the botanical point of view, some of these terms could be grouped as variants of the organs: stem, fruit and seeds.

Considering the number of citations for each vegetative/reproductive organ noted by Marcgrave, among the consumed parts of the plant, the fruits were the most cited organ in the book of reference (55 citations), followed by seeds (twelve citations), roots (seven citations), leaves (six citations) and nuts (three citations), and the other parts had one citation each (Table 1).

Regarding the mode of consumption of these food plants, Marcgrave mentions 20 modes of ingestion, listed below according to their citation frequencies in the work: cooked (22 citations); fresh, considering for this term the direct ingestion of fruits, seeds and pulp [22]; grilled (five); wine (five), crude (four) as drink and beverage/juice (three citations each); vinegar, honey, spice, dried and conserve (two citations each); mellow, salad, fried, oil, flower, production of sugar and with sugar and seeds in a dough (one citation each) (Table 1). Notably, in addition to the parts of the plant used, some modes of consumption could also be grouped. Thus, it would be possible to consider as fresh usage types the following variants: raw, dried, mellow, salad and spice.

The modes of consumption are diverse and allow the identification of some relation with the part of the plant used. Taking as an example the use of the fruits of the “umari” (*Geoffraea* sp. - Fabaceae), the indicated mode of ingestion was cooked (Table 1). It is interesting to note that Marcgrave himself said that the fruit of this plant if “eaten raw is prejudicial to the stomach and causes vomiting; that is why it is cooked and smashed with the core” ([36], p. 121). It is noticeable that for some species, there was an appropriate mode of consumption that would avoid the action of chemical constituents of the plant with harmful effects to health or even that would allow better digestion and/or palatability of the consumed part. Commenting on the flavour of Brazilian fruits, the French botanist Auguste de Saint-Hilaire also reveals this need to improve the palatability of some species by adopting appropriate modes of consumption. Saint-Hilaire states that throughout the different natural environments of Brazil, a huge quantity of fruits could be eaten with pleasure, provided “some care” was given to them; otherwise, the fruits from Europe would be tastier than the ones from Brazil [50]. Another example related to human health is in the therapeutic indication associated to the consumption of the fruits of the pineapple (*Ananas sativus* Schult. & Schult. F. - Bromeliaceae), whose fresh ingestion would be “given to the sick” ([36], p. 33). The pineapple is considered a functional and nutritious food, rich in vitamin C, beta-carotene (provitamin A), B vitamins and minerals such as potassium, manganese and calcium, in addition to the enzyme bromelain. This fruit has the function of restoring energy, has an antispasmodic effect, is a mild laxative and diuretic and has a moderate antioxidant property [51]. Linked to this therapeutic indication, there is evidence that the fruits represent a rich source of vitamins, minerals and fibre [52,53], which would accelerate the recovery of a patient and/or prevent the establishment of disease. Another aspect related to the consumption of fruits is indicated in the writings of Spix and Martius [54], who, when describing their trip through the backcountry of the state of Bahia, noted the use of the fruits of some plants to refresh them and their thirsty animals.

Regarding the origin/ethnicity of the social actors that Marcgrave indicated as using a certain plant as a food resource, although this information was not present in the comments for all the plants considered by Marcgrave, indigenous and African brazilians were the groups most associated with the use of these plants. In total, 32 citations were registered, referring to six ethnic groups that used the food plants referenced by Marcgrave, with 22 citations of use related to Indians, five to blacks and one citation each for the Portuguese, the “people of Guinea”, Ethiopians and Americans (Table 1).

Analysing the historical trajectory of these species according to their indications of consumption by specific ethnic groups, it is noticeable that the introduction of some of these plants to Brazil occurred only through the ethnic groups described by Marcgrave. This pattern was the case of *Sesamum orientale* L. (Pedaliaceae), in Marcgrave's popular designation “sesame”, “gangila” or even “girgilim”. Originating from Indonesia, China, India and tropical Africa, the plant has been cultivated in India since 1600 BC. African slaves brought their seeds from Africa to the Americas. In line with this history of transportation of “girgilim”, Marcgrave identifies the blacks as consumers of the oil extracted from its seeds. Commenting on the culinary uses of this plant, Felippe [55] states that sesame is used for oil production. Sesame oil is a key ingredient in Arabic food and tahini sauce, in addition to its seeds being traditionally used in the preparation of breads, cakes, etc. Regarding the past use of this plant, Felippe [55] states that in Northeast Brazil, at the time of slavery, a toasted sesame flour mixture had a widespread use.

“Perexil” (*Iresine vermicularis* (L.) Moq.) also has an indication of consumption in concordance with the ethnic group that was responsible for its introduction to Brazilian lands. Regarding the use of this plant food, Marcgrave states that “the leaves and branches cut short and boiled with a little vinegar can be spiced and preserved as a pickle, to be eaten with meat and fish. It has a great flavour and is highly valued by the Portuguese” ([36], p. 14). Henriquez, physician to King John V of Portugal (1689-1750) states in his treatise that “in order to conserve a healthy life”, “perexil” was eaten as a condiment in Portugal [56].

Marcgrave, commenting on the consumption of the root of the yam or “yam of São Tomé” (*Polynome alata* (L.) Salisb.), states that it was a food that replaced bread for the “inhabitants of Guinea”. Combining this information with the consumption data of this plant presented by Felippe [55], processed tuberous roots were one of the foods most appreciated by the blacks from West Africa. The yam is a plant from Vietnam, which had a wide distribution in prehistoric times, including other species of the genus [55].

Based on an analysis of each ethnic group and the parts and mode of ingestion of food plants, Indians notably account for the majority of consumption of fruits (16 citations) eaten fresh and/or “mellow” (five citations); as spice, juice, wine or conserve (with a citation each); or with an unspecified mode of consumption (seven citations). Among the fruits consumed fresh, examples include the pineapple, the pepper (*Capsicum annuum var frutescens* (L.) Kuntze - Solanaceae) and “inga” (*Inga vera* Willd. - Fabaceae). The mode of consumption could be a way that the Indians found to minimise or even remove the toxic effects present in some of the species consumed. However, it was not possible to establish a relation between the part of the plant and its mode of consumption. Regarding other ethnic groups, it was not possible to trace a tendency of preference. But, according to the concise narrative of Marcgrave it is possible to assert that the blacks consumed a large variety of plant parts, such as the root, leaves, flowers, fruits and seeds. The author points the use of “meeru” (*Canna indica* L. - Cannaceae), where Marcgrave says that “the blacks eat the root” (HNB, p. 4) (see Table 1). For “pindoba” (*Attalea oleifera* Barb. Rodr. - Arecaceae) and “sésamo” (*S.orientale*), there is a more detailed report, that is that these species were consumed by the blacks as a pulp, eaten with flour and as an oil “extracted from the seed, and residuals eaten with corn” ([36], p. 133-134; p. 21).

### Considerations of the use of Marcgravian species today

By analysing the knowledge/use of species mentioned by Marcgrave over time, a relation between historical and current uses may be observed. For some plants, such as the “umbu” (*Spondias tuberosa* Arruda) and “pitomba” (*Talisia esculenta* (St. Hill.) Radlk.), the knowledge/recorded usage in the 17th century source was maintained over the centuries in the northeast region of Brazil [13,57,58]. Other species are also currently consumed in other regions of the country and in various regions of the world. This pattern was the case of plants such as guava (*Psidium guajava* L.) and papaya (*Carica papaya* L.), which have a wide geographical distribution and are traded in the international market of food products [55,59]. Rich in vitamins B and C, the guava is a plant that originated in tropical America, southern Central America and northern South America [60]. The earliest evidence of guava cultivation in Mexico dates from the beginning of the Christian era. The introduction of guava to Brazil occurred before the colonial discovery, and the Portuguese took it to their other colonies [55]. The papaya originated from Panama and the northwest coast of Colombia and was taken to Malaysia, the Philippines and other countries of the East between the 1600s and 1700s. The latex, seeds, leaves, stem bark and green fruits of papaya contain papain, a proteolytic enzyme used for the digestion of meat. The fruits are rich in vitamin C and A and are currently consumed fresh and as juice, ice cream, jelly and candied fruit [55,60].

Some species deserve special attention because they are endemic plants of the Caatinga region, comprising the Brazilian semiarid region. This group includes the aforementioned “umbu”, the “mandacaru” (*Cereus jamacaru* DC.) and the “carnauba” (*Copernicia cerifera* Mart.).

“Umbu” has been reported over time as a central species for feeding the local population. In addition to Marcgrave, other naturalists also recorded its food usage even before their passage through the region, such as Gabriel Soares de Sousa in 1587 [61] and also after the period that he spent in Brazil in the 19th century, as can be seen in George Gardner's writings in 1846 [62] and Philipp von Martius's in 1844 [63]. Papers published in the mid-20th and early 21st century also indicate the current consumption of the fruits of this plant, mainly fresh [13,57,58,64,65]. Another mode of preparation for the consumption of fruits, considered characteristic of the backlanders (sertanejos), was referenced by Martius [63]: the food traditionally called “umbuzada”, classified by the naturalist as a healthy and refreshing food. Currently, the consumption of “umbu” fruits is diverse, in the form of beverages, ice cream, jams, jellies, vinegars, wine and liquor [57]. Moreover, salads are also prepared with the green or fresh leaves of the plant, and edible flour is produced using its root [57]. The backlanders also uses the root to relieve thirst and hunger, consuming the root in the same way as they would sugarcane [66]. Within this vegetable organ, studies have found the presence of protein, crude fibre, ether extract, tannin, starch, sulphur, phosphorous, calcium and magnesium [67,68].

With an equal food indication, “mandacaru” is mentioned by Marcgrave and Andrade-Lima [64], who describe the consumption of fresh fruits. Evaluating the physicochemical properties of the fruits of this species, studies indicate the potential for exploitation in industry and biotechnological processes, including alcoholic and acetic fermentation and the production of distilled drinks [69,70].

For “carnauba”, in addition to Marcgrave, Arruda Câmara [71] and Braga [66] commented on the ingestion of “carnauba” root as a condiment and of the fruits, nuts and heart of palm in a fresh form. However, for this species, there are no current studies addressing the issue of nutritional potential.

The list of food plants presented by Marcgrave was represented in 18 ethnobotanical studies conducted in Northeast Brazil (Table 2). In these works, there was information pertaining to 33 species with food uses in the referenced primary source (28% of the total species). However, for this group of plants, the data from current research were not restricted to food usage, with indications of use in other categories such as medical use, fence construction, shade and forage production (see, e.g., 13 and 72). Concentrating the analysis only on species indicated for consumption today, there is a reduction in the number of food plants to 11 (see Table 2). Thus, for example, it can be asserted that the use of species such as *Anacardium occidentale* L., *Crateva tapia* L., *Manihot glaziovii* Müll. Arg., *P. guajava*, *S. tuberosa* and *T. esculenta* were perpetuated over the centuries through the habits of the population of the northeast region of Brazil [13,16,72,79-93].

**Table 2:** Marcgravian edible species mentioned in ethnobotanical studies conducted in the northeast region of Brazil

**Taxonomic track**	**Popular name (Local)**	**Use category (PU)**	**Reference**
**Anacardiaceae**			
*Anacardium occidentale* L.	Caju-roxo, Caju-branco, Cajú, Cajueiro (RNE - PE, PB, CE)	Fo, Med (Bar, Le), Woo, Fue, Cons, Tec, FC, SP	[79-91]
*Schinus terebinthifolius* Raddi	Aroeira (RNE)	Med (Bar), Woo, Fue, Cons, Tec	[79,82,90]
*Spondias purpurea* L.	Seriguela, Siriguela (RNE - PE, CE)	Fo, Med (Bar, Le), Woo, FC	[72,79,81,82,85,86]
*Spondias tuberosa* Arruda	Imbu, Umbu, Umbú, Umbuzeiro (RNE - PE, PB)	Fo (Fr, R), Med (Bar, Le, Fr, R, Re), For, Woo, Fue, SP	[13,72,79-81,83-85,87,89,92]
**Annonaceae**			
*Xylopia frutescens* Aubl.	Imbira-vermelha (PE)	Woo, Fue, Cons, Tec	[82,90]
**Arecaceae**			
*Cocos nucifera* L.	Coco (RNE - PE)	Fo, Med (En), Woo, Cons, Tec, FC, Orn, SP	[72,79,81,82,85,86,90]
*Syagrus coronata* (Mart.) Becc.	Licurizeiro (RNE)	Med (Fl, Fr)	[79]
**Bignoniaceae**			
*Crescentia cujete* L.	Coité (RNE - PE)	Med (Le, S)	[79,91]
**Bixaceae**			
*Bixa orellana* L.	Urucum (RNE - CE)	Med (Le, Fr, S)	[79,88]
**Bromeliaceae**			
*Ananas sativus* Schult. & Schult. f.	Abacaxi (RNE - PE, CE)	Med (Fr)	[79,81,88,89]
**Cactaceae**			
*Cereus jamacaru* D.C.	Mandacaru, Mandacarú, Babão, Cardeiro (RNE - PE, CE)	Fo (P, S); Med (Cla, Fr, R)	[15,79-81,83,85,88,91]
**Cannaceae**			
*Canna indica* L.	Cana-da-índia (RNE)	Med	[79]
**Capparaceae**			
*Crataeva tapia* L.	Trapiá (RNE - PE)	Fo, Med (Bar, Le, Fr, R), Fue, Cons, Tec, For, SP	[72,79,84,91,93]
**Caricaceae**			
*Carica papaya* L.	Mamão, Mamoeiro, Papaya, Mamão de corda (RNE - PE, CE)	Med (Fl, Le, Fr, La), Woo, FC	[79-82,85,86,88]
**Convolvulaceae**			
*Ipomoea batatas* (L.) Lam.	Batata-doce (CE, PE)	Med (Le)	[80,85,88]
**Cucurbitaceae**			
*Cucurbita pepo* L.	Jerimum, Abóbora (RNE)	Med (S)	[79,81]
*Citrullus lanatus* (Thunb.) Matsum. & Nakai	Melancia (PE)	Med	[85]
**Euphorbiaceae**			
*Manihot esculenta* Crantz	Mandioca (RNE - PE, CE)	Med (Le, La, R)	[79,88,89]
*Manihot glaziovii* Müll. Arg.	Maniçoba (RNE)	Fo (R), Med (Fl, Le)	[16,79]
**Fabaceae**			
*Cajanus cajan* (L.) Huth	Feijão guandu, Feijão guandu (PE)	Med (S)	[80,81,91]
*Lablab purpureus* (L.) Sweet	Feijão cabricuço (PE)	Med (S)	[91]
**Malvaceae**			
*Hibiscus esculentus* L.	Okra (PE)	Med (Fr, S)	[91]
**Musaceae**			
*Musa paradisiaca* L.	Banana-prata, Banana (RNE - PE, CE)	Fo, Med (Le, Fl, Fr, R)	[72,79-81,85,88,89]
**Myrtaceae**			
*Eugenia uniflora* L.	Pitanga (RNE - PE)	Fo, Med (Le), Woo	[72,79-82,85]
*Psidium guajava* L.	Goiaba, Goiabeira, Goiaba branca (RNE - PE), Goiaba vermelha (CE)	Fo, Med (Le, Fl, R), Woo, Fue, Cons, Tec, SP	[72,79-83,85,88-90]
**Passifloraceae**			
*Passiflora cincinnata* Mast.	Maracujá-do-mato (CE)	Med (Le)	[88]
**Pedaliaceae**			
*Sesamum orientale* L.	Sesame, Gergelim-branco (RNE - PE)	Med (S)	[79,91]
**Piperaceae**			
*Piper marginatum* Jacq.	Capeba (RNE - PE)	Med (Le)	[79,91]
**Portulacaceae**			
*Portulaca oleracea* L.	Beldroega-branca, Bedroégua (RNE - PE)	Med (Le, AP), For	[79,92]
**Rubiaceae**			
*Genipa americana* L.	Genipapo (RNE - PE, CE)	Med (Bar, Fr), Tec	[79-81,88,90,91]
**Sapindaceae**			
*Talisia esculenta* (A. St.-Hil.) Radlk.	Pitomba (PE)	Fo, Med (Bar), Woo, Fue, Cons, Tec, FC, SP	[72,80-82,84-86,90,91]
**Solanaceae**			
*Solanum agrarium* Sendtn.	Melancia-da-praia, Gogóia (RNE)	Med (WP, R)	[79]
**Xanthorrhoeaceae**			
*Aloe vera* (L.) Burm. f.	Babosa, Erva-babosa (PE)	Med	[89]

Legend: RNE = Northeastern Region; PE = State of Pernambuco; PB = State of Paraíba; CE = State of Ceará; Fo = food; Med = medicinal; Woo = wood; Fir = firewood; Fen = fence; Fue = fuel; Cons = construction; Tec = technology; FC = fence construction; For = forage; Orn = ornamental; SP = shade production; PU = part used (R = root; Fr = fruit; P.Fr = pseudofruit; En = endosperm; P = pulp; S = seed; Le = leaf; Fl = flower; Bar = bark; I.Bar = intra-bark; Re = resin; La = latex; Cla = cladode; AP = aerial parts; WP = the whole plant).

The part of the plant ingested is not specified in much of the current research. However, for *S. tuberose*, there is only mention of the consumption of the fruit and root of this plant [13]. In *Historia Naturalis Brasiliae*, however, Marcgrave states that in addition to these organs, the leaves of “umbu” were used in a beverage (Table 1).

How can one justify the small number of plants with indications of current food use in relation to the total list of Marcgravian food species?

Two factors may have been crucial to generate this scenario: the focus of the analysed research was not exclusively to record the food use of plant species, and works with different approaches were included because few studies were dedicated solely to food plants; additionally, there may be weaknesses in the methodology of works devoted to the investigation of food plants.

It would be possible to also consider a historical view of the dynamics of knowledge and/or use of plant species in human cultural practice. From this perspective, the food use of a plant would serve as a starting point for the species to be integrated into the local culture. As part of the diet, this plant would be subsequently and gradually included in other categories of use, and new applications would go through cultural fixation with the passing of time. In this process, the act of feeding upon this plant would lose its importance, and the food use would no longer be a habit remembered and transmitted among the members of a given human community. This hypothesis may be reinforced by the fact that a high percentage of Marcgravian species are used today in categories other than food (Table 2).

Among these categories of current use, the medicinal category stands out (32 species) (Table 2). In a study discussing the ways by which exotic species enter the knowledge and practices of local populations, it is stated that these species are used for various purposes, such as for food and ornamentation [73]. This study proposes the idea of versatility, which has been assumed as a hypothesis by other authors [74], asserting that medicinal use would not be the reason for a species’ introduction into a given culture; rather, the therapeutic use would develop in a second phase. An exotic species with greater versatility has a greater likelihood of inclusion within cultural habits and subsequent medicinal use. Contemplating this scenario presented by the versatility hypothesis and the presence of 17th century plants listed in the contemporary literature primarily for medicinal use, it is suggested that food use has functioned as a “gateway” for plant resources (native and/or exotic) into the culture of the northeast region of Brazil. Thus, the food species described by Marcgrave would have been incorporated by the local pharmacopoeias over time. In a broader analysis of Brazilian culture, it is possible to consider that certain species may have become medicinal plants and ceased to be recognised as food due to the commonly present attitude among the Brazilian population of not considering food as good for health but seeking cures for illnesses through medicines. In this case, the transition from the food category to the medicine category would be explained by this mentality of giving little importance to food as preventive medicine. Only in recent years has this situation begun to change through the dissemination by the media of the idea that a balanced diet is associated with individual good health and wellbeing.

It must also be considered that local populations, particularly those settled in the northeast region of Brazil, began to associate certain plants with periods of food scarcity. It is common for local populations to consider the consumption of species that entered the diet in extreme periods of food shortage shameful [75]. For local populations throughout Latin America, this attitude of considering the ingestion of these plants a sign of poverty is present [76,77]. This attitude may help to elucidate the process of the use/disuse of plant species as food sources. The cultural taboo associated with a plant food would then be an imperative factor in the restriction and/or gradual discontinuation of the use of certain resources. Cultural taboos become decisive in the erosion process of local knowledge because certain “knowledge” and “practices” become unprivileged information during the oral transmission of knowledge and cultural practices, a mode of transmission commonly exercised in human cultures [78]. As a result of this disruption, the knowledge lacks subsequent perpetuation among generations of local populations.

In the quest to understand the complex processes of the arrival, fixing and/or deletion of food plants in the diet of the human population of a particular place, it is essential to refer to the story. Historical analysis serves as a facilitating approach to unravel Brazil's multicultural diet, translating and identifying peoples, cultures and eating habits that were enriched and adapted during the development of the country.

## Conclusions

In his treatise *Historia Naturalis Brasiliae*, Marcgrave mentions a diverse cast of food plants, mainly of arboreal habit. In a thorough manner, the characteristics of each species are revealed along with what they represented for Indians, Africans and Portuguese – the ethnic groups who held the knowledge of their use.

Despite the continued presence of the food species described by Marcgrave in the regional food habit, and even on a global scale, there has been a lack of studies of some species endemic to the Caatinga for exploitation in biotechnological processes and agro-industry. There is, therefore, a pressing need to devote efforts to deepen the knowledge and use of food species native to this region. The great nutritional and productive potential of these species have been undervalued, losing the opportunity to meet the demand for local consumption and even the demands of the global market that could exist if emphasis was given to the study of these species. Useful future research could be conducted considering species such as *C. jamacaru* and *C. cerifera*.

The analysis presented here allows a transfer of information about species used for food from the past to the present. It is hoped that future studies promote a change in the specialised dietary patterns of today, based on the research presented here. Finally, a means of food diversification, accessible to local populations, may be achieved through the use of this information for the development of new food products.

## Competing interests

The authors declare that they have no competing interests.

## Authors’ contributions

UPA and MFTM contributed to the design of the study, interpretation of the findings and preparation of the manuscript. Both authors read and approved the final manuscript.
